# Protocol for the effect evaluation of independent medical evaluation after six months sick leave: a randomized controlled trial of independent medical evaluation versus treatment as usual in Norway

**DOI:** 10.1186/s12889-017-4469-3

**Published:** 2017-06-14

**Authors:** Elisabeth Husabo, Karin Monstad, Tor Helge Holmås, Irene Oyeflaten, Erik L. Werner, Silje Maeland

**Affiliations:** 1 Uni Research Health, PB 7810, 5020 Bergen, Norway; 2 Clinic of Child and Adolescent Mental Health, Haukeland University Hospital, PB 1400, 5021 Bergen, Norway; 3 Uni Research Rokkan Centre, PB 7810, 5020 Bergen, Norway; 4 National Centre for Occupational Rehabilitation, Haddlandsvegen 20, 3864 Rauland, Norway; 5 Department of General Practice, Institute of Health and Society, University of Oslo, PB 1130, Blindern, 0318 Oslo, Norway; 6 Research Unit for General Practice, Uni Research Health, PB 7810, 5020 Bergen, Norway; 7 Department of Occupational Therapy, Physiotherapy and Radiography, Faculty of Health and Social Sciences, Bergen University College, Norway, PB 7030, 5020 Bergen, Norway

**Keywords:** Sickness absence, Sickness benefits, Primary care, General practitioner, Family physician, Social insurance, Randomized controlled trial, General practice, Disability, Disability pension

## Abstract

**Background:**

It has been discussed whether the relationship between a patient on sick leave and his/her general practitioner (GP) is too close, as this may hinder the GP’s objective evaluation of need for sick leave. Independent medical evaluation involves an independent physician consulting the patient. This could lead to new perspectives on sick leave and how to follow-up the patient.

**Methods/design:**

The current study is a randomized controlled trial in a Norwegian primary care context, involving an effect evaluation, a cost/benefit analysis, and a qualitative evaluation. Independent medical evaluation will be compared to treatment as usual, i.e., the physicians’ and social insurance agencies’ current management of long-term sick-listed patients. Individuals aged 18–65 years, sick listed by their GP and on full or partial sick leave for the past 6 months in Hordaland county will be included. Exclusion criteria are pregnancy, cancer, dementia or an ICD-10 diagnosis. A total sample of 3800 will be randomly assigned to either independent medical evaluation or treatment as usual. Official register data will be used to measure the primary outcome; change in sickness benefits at 7, 9 and 12 months. Sick listed in other counties will serve as a second control group, if appropriate under the “common trend” assumption.

**Discussion:**

The Norwegian effect evaluation of independent medical evaluation after 6 months sick leave is a large randomized controlled trial, and the first of its kind, to evaluate this type of intervention as a means of getting people back to work after long-term sickness absence.

**Trial registration:**

ClinicalTrials.gov NCT02524392. Registered June 23, 2015.

## Background

Long-term sickness absence (LTSA) may lead to exclusion, weakening of economic independency and create dependency of health services, welfare services and/or social welfare. The majority of individuals with illness and disease do not need any assistance to return to work (RTW) after a period of sick leave [1, 2]. However, approximately 6.5% will still receive sickness benefits after 6 months [3].

In Norway, 330 individuals reach 6 months of uninterrupted sick leave every day. For these individuals, work disability may lead to disability pension [3]. Amongst 15 OECD countries, Sweden and Norway had the highest percentage increase in people between 20 and 64 years on disability pension, from 1980 to 2007/2008 [4]. This is a negative trend, because very few return to work after having received disability pension [3].

The Norwegian Labor and Welfare Administration (NAV) is the public welfare agency in Norway. It is responsible for a third of the state budget, administering state and municipal welfare agencies, including sickness benefits, pensions, unemployment benefits and social security benefits. Workers are entitled to sickness benefits from NAV if they have been in paid work for the last 4 weeks before the sickness incident. In general, employees receive 100% of their salary (up to 6 G = 555,456 NOK, approx. $ 67,501 in 2016) in sickness benefit from the first day of reported sick and up to 1 year. The employer pays for the first 16 days of a sick leave period, and thereafter NAV covers the disbursement.

The current sickness absence management is organized by NAV. Within 4 weeks from the sick leave starts, the employer is supposed to create a follow-up plan and within 7 weeks arrange a meeting with the sick listed employee. NAV and/or the GP may be invited. The goal of this meeting is to find solutions for RTW, secure dialogue and update the follow-up plan. The employer has to provide work-related activity within the first 8 weeks of sick leave. At 26 weeks of sick leave, NAV arranges for a second meeting, where the person on sick leave and the employer are obliged to participate. The purpose is to find solutions for RTW and to secure a shared view of responsibility and time line.

Sick leave can be graded from 20 to 99% independent of the proportion of employment; full or graded. If the employee does not RTW after 1 year, he or she may receive a transition benefit, which has an upper limit of 4 years. NAV’s general goal is to have more people working and fewer on benefits.

The GP is an important stakeholder, issuing 70% of all sickness certificates in Norway [5]. Most GPs in Norway are self-employed, but are registered on a public list-based GP-scheme with a contract through the local authorities. GPs have no direct financial incentives regarding the length of their patients’ sick leave. Since the GP-scheme entitles all citizens to have a personal GP, the GP is assigned an important role in RTW efforts. Citizens can change GP twice a year. The GPs are reimbursed by a fixed yearly allocation of 380 NOK per patient on the GP-scheme and the average GP list size is 1200 people [4, 6]. Additional reimbursements include consultation fees from patients and the health authorities, based on fixed tariffs.

The patient-GP relation is discussed as problematic by several interests [7]. It is debated whether the public list-based GP-scheme has increased the competition between GPs, and made them more concerned with keeping patients satisfied, than with being strict gatekeepers [8, 9].

Independent medical evaluation (IME) involves an independent physician consulting the patient. It is traditionally requested by insurance companies, attorneys, case managers, and employers who want to establish “estimated” physical capacities, degree of disability, and the reason for work disability [10]. It is also used when there is uncertainty around the functional status and/or the employee’s rehabilitation potential. Ethical and legal issues of IMEs have been discussed, especially the detached relationship between the IME doctor and a vulnerable patient [11].

IME is not commonly used in Norway. The most similar parallel to an IME in a Norwegian context is when NAV is unsure about the GP’s evaluation of the patient’s rights to disability pension and work assessment allowance. A physician employed by NAV then looks at the patient’s medical records and makes recommendations about imbursements. However, this procedure is usually introduced when a patient’s sick leave reaches 1 year. The purpose and organization of the IME of this RCT is outlined below.

In this paper we present the protocol for an ongoing RCT, which aims to explore whether an IME at 6 months of sick leave increases the RTW rate of long-term sick listed patients, compared to the current management of these patients.

## Methods/design

### Aims and objectives

The main aim of this study is to evaluate the effect of IME compared to TAU. The intervention is given to patients after 6 months of continuous sick leave (full or graded sick leave). The IME is conducted by specially trained GPs, performing the IME consultation at their own GP practice in the Norwegian county Hordaland. The following questions will be addressed:

- Is IME more effective than TAU in getting people back to work after 6 months of sick leave (measured in sick leave grading and length of absence)?
- Does IME affect sick leave in the Hordaland county control group, compared to sick listed in other control counties, appropriate to comparison?
- Is IME cost-effective compared to TAU from a societal perspective?
- How is the intervention affecting the involved stakeholders?

### Outcome measures

The primary outcome is change in sickness benefits at 7, 9 and 12 months. This will be identified through register data from NAV, The Norwegian Health Economics Administration (HELFO), and Statistics Norway.

### Data collection

Data collected throughout the project is saved in NAV’s secure online database. All directly identifiable personal data (name, social security number, address etc.) is replaced by a unique ID-number before data are sent to Uni Research twice during the project period. Complete and objective data are collected from NAV’s national social insurance register, with no loss to follow-up. Administrative costs at NAV, the IME-physicians’ salary, office rent and patient travel costs will be used for the cost/benefit analysis. Information about education level will be obtained from Statistics Norway, which has the responsibility for official statistics in Norway. HELFO, which is a sub-ordinate institution to the Norwegian Directorate of Health, will provide information about the GPs.

### Study design

This RCT comprises 1) an effect evaluation, 2) a cost-benefit analysis, and 3) a qualitative evaluation. The main focus of the study is the effect evaluation. The cost-benefit analysis is included to evaluate the resources invested in the intervention, compared to the potential economic gain from reduced sickness absence and increased labor market activity. The qualitative evaluation is conducted to gain insight into stakeholder’s experiences.

### Effect evaluation

**Fig. 1:**
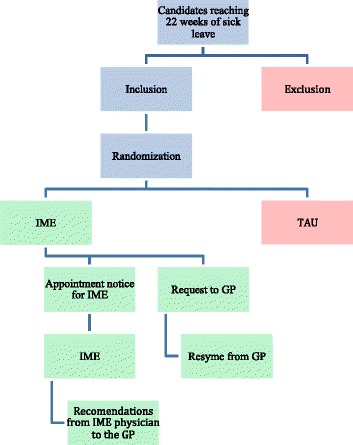
Study flow

The effect evaluation is a RCT, which is ideal for the aim of comparing interventions to increase RTW of sick listed individuals. See Fig. 1 for study flow.

### Interventions

The procedure is organized by NAV. By a randomization procedure, eligible participants are allocated to the IME or TAU. The IME procedure is developed in cooperation with the Research Unit for General Practice at Uni Research Health, and NAV.

Two weeks in advance of the IME consultation, NAV sends an appointment notice to the IME participant, and a request to his/her personal GP to send a medical summary to inform the IME physician. The GP is payed a fixed rate for this summary. The IME physician has a total of 2 h for preparation, consultation (30–60 min) and for writing an IME report. The IME report is sent to the patient, the patient’s GP and NAV. The report will primarily serve as a tool for the GP’s continued follow-up of the patient. The IME report provides a second opinion on the patient’s fitness for work or need for continued sick leave, full or graded. The GP may use this evaluation in his/her communication with the patient, particularly if the IME report points at new options for treatment or other initiatives. The GP is responsible for further follow-up actions and sick leave certificates. Both the intervention group and control group receives TAU (regular follow-up in Norway), which is described in the background section. There are no restrictions in participation in concomitant care or interventions during the study.

Specially trained GPs (the IME physicians) are temporarily employed by NAV to perform the IME consultations. The IME physicians were recruited through job announcements. GPs were required to have a minimum of 3 years clinical practice, with completed or ongoing specialization in general practice. As a main rule, the IME physicians use their own offices for the IME consultation. The training of the IME physicians consisted of a 1-day course (7 h) with information about the project, lectures on basic risk factors for LTSA and the GP’s role in the sickness certificate process. The most important themes were addressed by casuistry and lectures of peers and social security stakeholders covering the complexity of illness, sickness, disease, functional ability, and work ability. Dilemmas of GP’s dual roles as gatekeeper and the patient’s advocate were discussed. Essential in the lectures were knowledge on health effects of working when sick, the Norwegian model of an inclusive working life, and the important role of the employer and the work place in relation to the sick-listed employee. In depth information about the Norwegian social security legislations and work-related actions within the NAV system was included in the lectures. Furthermore, the IME physicians learned about the specific procedures necessary to perform the IME. This included training in using the NAV database in order to get access to information about patients. A second, 3-h training session for the IME physicians was held after a 4-week pilot-period. IME physicians participating in the pilot shared their experiences with the other IME physicians and they all received training in practical and technical procedures.

A third, 4-h training session with the IME physicians was held 4 months after project start. Prior to this, two specialists in the field assessed three IME reports for each of the physicians, and gave written, specific feedback. At the third session the feedback was discussed with the IME physicians individually. Rest of the session was used for group discussions.

As a framework for themes addressed in the IME consultation, the IME physician use a report form. Before meeting the patient, the IME physician prepares for the consultation by starting to fill out the report form with background information from the NAV database and the GPs summary. The IME report includes the following information:

- Name of IME physicians, date of consultation, name of patient, national identity number and a check box for whether the patient did meet or not for the IME consultation.
- Name of the responsible NAV office, name of the patient’s GP and a check box for whether the GP did send a medical summary or not in advance of the IME consultation.
- Brief background information of the patient’s case history: Date of when the current sick leave started, any specialist report enclosed and grade of sick leave at the time of the IME.
- Information from the patient on work-related actions carried out during the current sick leave period (partial work, “a faster RTW” - actions, dialogue meetings, other work-related actions).
- The patient’s resources towards RTW (having an employer, type of occupation, type of work tasks, manageable work tasks for the time being, manageable work tasks if adjustments at the workplace, manageable tasks at home).
- The patient’s own descriptions of why the work ability is reduced related to medical reasons (somatic, mental, cognitive), practical reasons (waiting for medical elucidations or treatment), specific work tasks at the current work place (tasks that can be done or not, and possibilities of adjustments), work tasks at the labor market in general (unemployed, competences).
- The patient’s own thoughts about RTW (full or partial), answered on a six-point check box (within a month, within 3 months, within the first year of sick leave, later, RTW is not likely (expected), no thoughts about RTW).
- The IME doctors evaluation of RTW (full or partial): What is the most important medical barrier for RTW (somatic, mental, cognitive factors)? What is the most important practical barrier for RTW? What is the mort important cognitive barrier for RTW? What is the most important barrier for RTW at the current workplace? Will a change of workplace with similar work tasks make it easier to RTW? Will a change of workplace with new work tasks make it easier to RTW?
- The IME doctor’s suggestion of actions, which must be justified (explained): Additional medical/psychological elucidation, additional medical/psychological treatment, actions/adjustments at the work place (contact NAV/employer), practical actions (job search, social security benefits), change of work place, dialogue meeting or other actions.
- The IME doctor must state whether sufficient and adequate actions have been accomplished until the current point of time.
- The IME doctor must suggest grade of further sick leave from the date of consultation (0–100%).
- The IME doctors concluding remarks, elaborations and summing up.

### Participants, recruitment, inclusion and exclusion

Participants aged 18–65 years old sick listed by their GP with an ICPC-2 diagnosis are recruited through NAV’s registries, when reaching 22 weeks of sick leave. All sick listed individuals are included, both those with and without an employer. Pregnant women, individuals with secret address, individuals employed by NAV, people with ICD-10 diagnoses (i.e., sick listed by the specialist health services), cancer or dementia are excluded. The goal is to recruit 1900 (1892) participants in each group, with a total sample of 3800 (3784) participants. Sick-listed in other counties serve as an extra control group, if comparison is appropriate under the “common trend” assumption. Included participants are exempt from participation when their treating GP explicitly states that the intervention could worsen the condition.

### Sample size calculation

According to NAV’s registry for Hordaland county, Norway, in 2008, only 15% of workers who had been sick listed for 6 months were not receiving sickness benefits at 7 months (Statistics Norway’s events database (FD-Trygd)). Table 1 shows sample sizes needed to estimate significant effects for five different effect levels (calculated in the analyze programme Stata, by the command “Power” with a 5% significance level, expected positive effect and power of 80%).

**Table 1:** Significance level. As scenario 3 shows, we will be able to discover an intervention effect of 3% if 1892 individuals in two groups participate in the study

	Expected effect level^a^	Sample size
Scenario 1:	Control group: 15% and IME: 16%	16,194 in each group
Scenario 2:	Control group: 15% and IME: 17%	4154 in each group
Scenario 3:	Control group: 15% and IME: 18%	1892 in each group
Scenario 4:	Control group: 15% and IME: 19%	1090 in each group
Scenario 5:	Control group: 15% and IME: 20%	714 in each group

Recent research shows positive effect of strict follow-up of people on sick leave in Norway [12, 13]. One RCT from Denmark [14] shows weak or no effect by increased follow-up on sick leave. These studies have been conducted on different groups, with different aims and measures different outcomes. Therefore, we cannot conclude on what can be viewed as a weak/moderate/strong effect. However, we do not expect a negative effect. This reduces necessary sample size compared to studies where the effect can be both positive and negative. We base our inclusion on scenario 3 (Table 1), and aim at recruiting at least 1900 participants to each group.

### Subgroups

Subgroup analyses of effects can be performed if sample size allows this. A priori, the following variables are particularly relevant for a subgroup analysis: age groups, gender, education, main diagnosis group and geographical location.

### Randomization

When registering eligible participants, trained personnel at NAV contacts a research technician at Uni Research Health by e-mail, stating only the participant’s ID-number, year of birth and gender. The participant is then allocated to one of two groups by use of a computer-generated randomization list.

### Qualitative evaluation

In order to investigate stakeholder’s experiences with the project, and how it affects them and the relation between them, two qualitative studies will be conducted. We will conduct semi-structured individual interviews with patients after attending an IME consultation and focus groups with GPs who have patients who attended an IME consultation. Topics discussed in the interviews and focus groups will relate to the stakeholder’s views of what changed/did not change the sick leave and/or follow-up after the IME consultation.

## Discussion

The effect evaluation of an independent medical evaluation (IME) after 6 months sick leave is a large RCT, providing evidence on the effect of IME on RTW after LTSA. It is the first controlled evaluation of IME.

## Abbreviations

GP: General practitioner
HELFO: The Norwegian Health Economics Administration
IME: Independent medical evaluation
LTSA: Long-term sickness absence
NAV: The Norwegian Labour and Welfare Administration
